# Emergency Laparotomy for Abdominal Compartment Syndrome in a Child due to Chronic Functional Constipation

**DOI:** 10.1155/crpe/5289632

**Published:** 2025-06-12

**Authors:** Konstantinos Velaoras, George Pantalos, Christos Plataras, Ioannis Alexandrou, Jonida Mene, Konstantinos Filos, Abhisekh Chatterjee, Panagiotis Nikolinakos, Nikolaos Zavras

**Affiliations:** ^1^Department of Pediatric Surgery, Penteli Children's Hospital, Athens, Greece; ^2^Department of Medicine, Faculty of Medicine, Imperial College London, London, UK; ^3^Department of Urology, Chelsea and Westminster Hospital NHS Foundation Trust, West Middlesex University Hospital, London, UK; ^4^Department of Pediatric Surgery, School of Medicine, Attikon University Hospital, National and Kapodistrian University of Athens, Athens, Greece

## Abstract

Abdominal compartment syndrome (ACS) in children is a life-threatening complication with high morbidity and mortality. Stressful life events are among the risk factors of functional constipation (FC) in children. We present a 13-year-old male patient with chronic FC due to parents' separation who presented with a history of FC since infancy and inability to defecate during the last month. On examination, the abdomen was distended and tender. His vital signs revealed elevated blood pressure ≥ 95^th^ percentile according to his age weight and gender. On admission, the patient experienced tonic–clonic seizures refractory to medical therapy. He was intubated and a computed tomography (CT) scan revealed an extensive rectosigmoid bowel dilatation. Despite maximal medical support, his condition worsened. ACS was suspected and confirmed via intravesical measurement of intra-abdominal pressure (IAP). An urgent decompression laparotomy (DL) was performed with resection of the affected bowel. His condition improved postoperatively. This case highlights the extremely rare association between ACS and chronic FC resulting from stressful life events.

## 1. Introduction

Abdominal compartment syndrome (ACS) is a life-threatening condition seen mainly in critically ill pediatric patients [1]. Any pathological event originating from an intra-abdominal process may elevate intra-abdominal pressure (IAP), affecting pulmonary, cardiac, hepatosplenic, renal, bowel perfusion, and central nervous function [2]. The exact incidence and mortality of ACS in the pediatric population vary among studies. In a recent study, an overall incidence of 0.17% and an overall mortality rate as high as 48.87% were reported [3].

Functional constipation (FC) is defined as constipation without an underlying organic, anatomical, or iatrogenic cause [4]. The prevalence of FC in children and adolescents varies according to geographical regions and age, but in general, it is estimated at 9.5% (0.5%–32%) worldwide [5, 6]. The condition becomes long-standing through a cycle of infrequent and painful bowel motions, fecal incontinence, stool withholding behavior, and occasional passage of large-diameter stools [7]. The pathophysiology of FC is unclear and seems to be multifactorial [8]. Risk factors associated with occasional or persistent FC include the child's psychosocial condition, withholding of stools after an experience of painful bowel movement, a slow transit constipation, genetics, and neurodevelopmental disorders such as autism spectrum disorders [7, 9]. An increasing body of literature has shown that exposure of children to stressful life events, such as bullying, sexual or physical abuse, and separation of parents or parental neglect, may be associated with chronic FC [8–10].

Herein, we report on a rare case of a child with chronic FC due to a traumatic life event who presented with ACS manifested with hypertensive crisis.

## 2. Case Report

**Figure 1:**
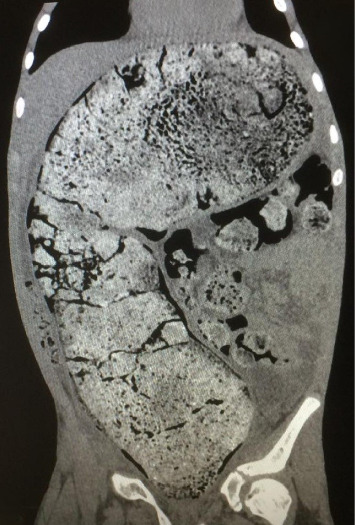
Coronal view of the abdominal CT scan, showing a distended sigmoid colon filled with large fecal masses with a maximum diameter of 11.58 cm.

**Figure 2:**
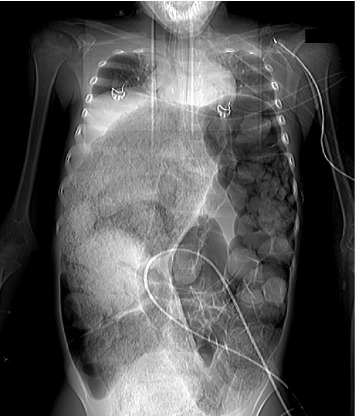
CT scanogram of the chest and abdomen showing severe bowel distension with fecal content, leading to elevation of both diaphragms and reducing the expansion of the lungs.

**Figure 3:**
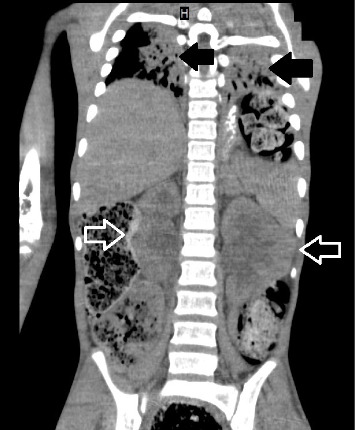
Coronal view of the abdominal CT scan, showing displacement of the liver and spleen, as well as significant dilatation of the renal pelvis and calyces due to bowel compression (white arrows). Note the restrictive bilateral lung compression with infiltrates (black arrows).

**Figure 4:**
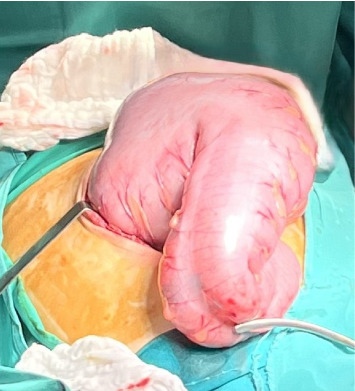
Intraoperative view showing a markedly distended rectosigmoid colon.

**Figure 5:**
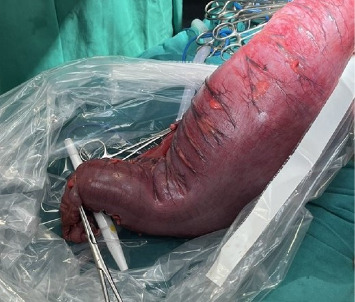
Intraoperative view of a markedly distended and ischemic rectosigmoid colon during Hartmann's procedure.

**Figure 6:**
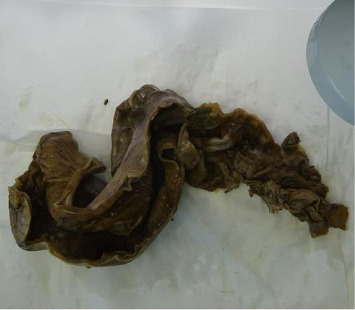
Macroscopic pathological view of the excised, markedly distended, and ischemic rectosigmoid colon.

A 13-year-old Caucasian male was referred to the emergency department of a rural secondary-care hospital in Greece due to chronic constipation and abdominal distention. His family history revealed that the child lived with his maternal grandfather since infancy after the divorce of his parents. An aversion to participate in any extracurricular or social activities was also reported by his caretaker. A chronic constipation state has been present since then, without other medical problems. The recent history uncovered a deficiency to defecate in the last month and intense abdominal distention during the last 2 weeks despite the use of laxatives. The patient, with a body weight of 41 kg (10^th^ percentile), and height of 141 cm (10^th^ percentile), was conscious on arrival, and initial examination showed a pale patient, with severe abdominal distention, rigidity on palpation in all quadrants, and absence of abdominal sounds on auscultation. The femoral pulses were not palpable. His initial vital signs were as follows: sinus tachycardia (heart rate: 160 beats/min), systolic/diastolic blood pressure (BP): 180/97 mmHg, respiratory rate: 20/min, capillary refill time: 2-3 min, and oxygen saturation: 95% on air room and afebrile. The examination of the rectum revealed a rectum filled with hard stools. Initially, four rectal enemas were administered and a large mass of stool was subsequently evacuated. The patient suddenly had an episode of tonic–clonic seizures which were managed with diazepam per rectum, followed by another attack of seizures refractory to rectal diazepam and intravenous (IV) midazolam. The pupillary light reflex was normal. He was intubated and transferred to the pediatric intensive care unit (PICU) of our tertiary pediatric hospital for further resuscitation. The initial blood tests were as follows: Hb: 7.7 g/dL (normal range: 12.0–14.0 g/dL), WBC: 29.600 μ/L (normal range: 3.5–9.5 × 10^3^ μ/L), platelets: 740.000 μ/L (normal range: 150–400 × 10^3^ μ/L), neutrophils: 94% (normal range: 40%–75%), and bandemia: 2%. Serological tests showed normal electrolytes levels, blood sugar: 131 mg/dL (normal range: 70–110 mg/dL), Ca: 8.9 mg/dL (normal range: 8.5–10.5 mg/dL), C-reactive protein: 16.5 mg/dL (normal range: 0–1 mg/dL), creatinine: 1.6 mg/dL (normal range: 0.5–1.35 mg/dL), and urea: 80 mg/dL (normal range: 10–60 mg/dL). The liver function tests were within normal limits. Blood gases demonstrated a normal pH: 7.43, pO_2_: 94 mmHg (FiO_2_: 45%), pCO_2_: 31 mmHg, HCO_3_: 22 mmol/L, and lactate concentration: 1.2 mmol/L. Coagulation tests (aPTT and INR) were normal (26.6 and 1.2, respectively). The insertion of an indwelling catheter yielded 20 mL of dark yellow urine. Urinalysis showed a specific gravity of 1030, ketones: ++, and proteins: 18 mg/dL. Plasma renin levels were markedly elevated: 49.7 ng/mL/h (normal values: 0.6–3.3 ng/mL/h). Thyroid function tests, catecholamines, and metanephrines were within normal ranges. He started antihypertensive therapy with labetalol, titrated according to the levels of BP. He received two units of pack red cells as well. A nasogastric tube was inserted for abdominal decompression and triple antibiotic therapy with metronidazole, amikacin, and cefotaxime was given to avoid sepsis. A chest X-ray revealed intestinal loops filled with fecal content, elevated bilateral diaphragms, and no free air in the abdomen (not shown). An echocardiogram showed hypertrophy of the left ventricle but a normal ejection fraction. After stabilization, a full-body computed tomography (CT) scan demonstrated no brain pathological findings. However, there was a remarkable distention of the total colon, especially of the sigmoid and rectum, with a diameter of 11.58 cm (Figure 1) and full of fecal content causing elevation of both diaphragms and reducing the expansion of the lungs, as they are shown in the full-body CT scanogram (Figure 2). There was a significant dilatation of the pelvis and calyces of the kidneys due to bowel compression; displacement of the liver, spleen, and stomach; and extrinsic compression of the inferior vena cava (Figure 3). The right renal pelvic anterior–posterior diameter was 1.7 cm, and the left one was 1.9 cm. Upon admission to the PICU, an intravesical pressure through the Foley catheter showed a markedly elevated IAP of 34 cm H_2_O, confirming our suspicion of ACS. The child was transferred to the operating theater for an urgent decompression laparotomy (DL). The surgical findings showed that the entire large bowel was distended with fecal masses. Upon entering the abdominal cavity and removing the distended colon (Figure 4), abdominal pressure was immediately relieved with ventilation improvement and increased urine production. A segment of the rectosigmoid colon (Figure 5), which was very dilated and ischemic, was removed and a Hartmann's procedure was performed. The pathological findings showed that the specimen (Figure 6), with a length of 48 cm and an inner diameter of 80 mm, presented thinning of the mucosal folds and extended distention of the lumen, with variable chronic ischemic lesions. The patient had an uneventful recovery, and the BP gradually improved over the next 5 days. The IV antihypertensive treatment switched to IV hydralazine and amlodipine per oral. On the 17^th^ day of hospitalization, he developed a fever of 38.5°C and abdominal pain. An abdominal CT scan revealed an intra-abdominal abscess which was treated by CT scan–guided percutaneous drainage.

During ward hospitalization, a rectal full-thickness biopsy was performed to rule out Hirschsprung's disease. Histopathological analysis showed the presence of normal ganglion cells without submucosal nerve hypertrophy, while the immunohistochemical markers were positive for calretinin and S100. Anorectal manometry revealed normal resting, squeeze pressures, and anal reflexes with no indication of dyssynergic defecation or internal anal sphincter achalasia. Other tests for celiac disease, cow milk protein allergy, and cystic fibrosis were normal as well.

While hospitalized, the child underwent psychological evaluation. Episodes of violent behavior and even physical abuse by the father were suspected to have occurred prior to the divorce of his parents. This was noted by the psychologists of our hospital after extensive discussion sessions with the child.

After 4 months of follow-up, renal function and BP returned to normal and oral amlodipine was discontinued. Urinary protein to creatinine and urinary calcium to creatinine ratios gradually returned to normal for age values. Accordingly, renal pelvic anterior–posterior diameters returned to 0.5 and 0.6 cm for the right and left kidney, respectively.

The patient received psychological support during hospitalization in our department and was discharged on the 22^nd^ postoperative day in good health. The transition of care was set up by the social services of our hospital so that the child can continue to receive psychological and primary-care support at his residence in a rural area. A closure of the colostomy is scheduled in the next 6 months.

## 3. Discussion

The term ACS in adult patients is defined as an IAP > 20 mmHg, leading to new organ dysfunction or failure [11]. However, in children, an IAP > 10 mmHg is considered as pathological [11]. For example, di Natale et al. reported a median incidence of IAP of 22.5 mmHg (range: 12–44 mmHg) in a population of 14 children undergoing DL for ACS [12]. In our patient, the IAP was 32 cm H_2_O and Grade IV according to the World Society of ACS [11]. Predisposing factors to ACS in children include gastroschisis, congenital diaphragmatic hernia, Hirschsprung's disease, peritonitis, intra-abdominal tumors (Wilm's tumor and Burkitt's), organ transplantation, burns, and septic or cardiogenic shock [13]. The clinical picture of pediatric patients with ACS is characterized most often by the deleterious effects of IAP on several organ systems, raising the need for urgent resuscitation [12–15]. Surprisingly, in the current case, the initial clinical presentation was characterized by a Stage II hypertension crisis [16], as defined by the American Academy of Pediatrics for gender, age, and height, expressed with refractory tonic–clonic seizures. We suspected that the elevated IAP caused a reduction in renal blood flow with a subsequent decrease in urine production, a rise in serum creatinine and urea, and a significant increase in plasma renin activity levels that eventually led to a hypertensive crisis. The hypertensive symptoms were resolved after DL, and BP returned to normal levels over the following postoperative days. It is of interest that Prasad et al. [17] described a closely related case of a child with chronic constipation who developed posterior reversible encephalopathy syndrome as a consequence of severe renovascular hypertension due to chronic fecal impaction. The symptoms subsided and the radiological findings resolved after aggressive clearing of fecal impaction.

The diagnosis of FC is based on Rome IV criteria [4] which include less than three stools per week, a minimum of one incident of fecal incontinence, a history of severe stool retention or retentive posture, a history of difficult or painful bowel movements, and a prior history of a large-diameter stool that could prevent the use of the toilet for at least 2 months. The exact pathophysiologic mechanism of FC in children exposed to stressful life events is not fully understood due to its complex nature [18]. It has been suggested that the brain–gut axis has a pivotal role in the pathogenesis of FC possibly because stressful life events reduce the ability of the central nervous system to refine visceral and somatic sensations [19]. The impact of parents' divorce on FC in children is not fully appreciated. The research [20] conducted in Indonesia found that separation of parents was a significant risk factor of FC in pediatric patients. Till now, there is no optimal therapy, and interventional strategies include several steps such as dietary interventions, education and behavioral therapies, fecal disimpaction, and new pharmacological interventions such as prosecretory, serotonergic, and bile agents; cholinesterase inhibitors; and transanal irrigation. [21]. Elective surgery has also been proposed with good results in selected cases [22]. Our literature review revealed 4 cases of ACS associated with FC, which were managed urgently with DL [23–26]. In 3 out of 4 cases [23–25], it seems that there was not a predisposing factor of FC, and in the final case [26], FC was related with the MECP2 duplication syndrome. This syndrome is a severe neurodevelopmental disorder including chronic gastrointestinal symptoms such as constipation and gastroesophageal reflux [27]. None of the aforementioned cases resulted in death. Our case links the FC of a child due to the stressful event of its parents' divorce with ACS.

In our case, DL was the last resort to resolve the hypertensive crisis and restore adequate ventilation and organs' perfusion. Our practice is supported by many authors [12–15, 28, 29], who propose DL as the definite treatment of ACS after the failure of medical management of elevated IAP.

This study underscores the importance of urgent recognition and early intervention with DL in the cases of intractable ACS. FC is a common condition that should not be ignored or left untreated as it can lead to disastrous complications. The association of prolonged fecal impaction with FC is rare and in this case that prolonged fecal impaction causing ACS is the most extreme harmful scenario. Furthermore, the study presented here poses the need to increase the awareness of pediatricians, caregivers, and psychologists in the cases of children with chronic FC and familial stressful events. Prevention and multidisciplinary treatment of FC are important, as it may progress from a stressful condition to a life-threatening one, as this case illustrates.

## Data Availability

No public dataset was used in the creation of this manuscript.
